# Effectiveness of Umonium^38^ against *Burkholderia pseudomallei, Escherichia coli, Pseudomonas aeruginosa* and Methicillin-Resistant *Staphylococcus aureus* (MRSA)

**DOI:** 10.1186/s12879-024-09102-9

**Published:** 2024-02-16

**Authors:** Soiratchaneekorn Ruanchaiman, Premjit Amornchai, Vanaporn Wuthiekanun, Sayan Langla, Peerapol Maroongruang, Khanh Kim Le, Stuart D. Blacksell

**Affiliations:** 1 Mahidol-Oxford Tropical Medicine Research Unit, Faculty of Tropical Medicine, Mahidol University, 10400 Bangkok, Thailand; 2 Centre for Tropical Medicine & Global Health, Nuffield Department of Medicine, University of Oxford, Old Road Campus, OX3 7FZ Oxford, UK

**Keywords:** Umonium^38^, Virkon, Bacterial viability, Disinfectant

## Abstract

**Aims:**

We investigated the antibacterial efficacy of Umonium^38^ and Virkon^®^ against *Burkholderia pseudomallei, Escherichia coli, Pseudomonas aeruginosa* and Methicillin-Resistant *Staphylococcus aureus* (MRSA) up to 14 days following treatment.

**Methods and results:**

Umonium^38^ was diluted to 0.5%, 1.0%, 1.5%, 2.0%, 2.5% and 3%, tested against the bacterial strains at various contact times (15 min to 24 h), and incubated for up to 14 days. A minimum concentration of 0.5% Umonium^38^ with a contact time of 15 min effectively killed approximately 10^8^ CFU/ml of all four bacterial species. No growth was observed on agar plates from day 0 until day 14 for all six concentrations. The bacteria were also inactivated by a 30-minute treatment time using Virkon^®^ 1% solution.

**Conclusions:**

Umonium^38^ effectively inactivates *B. pseudomallei, E. coli, P. aeruginosa* and MRSA at a concentration of ≥ 0.5% with a contact time of at least 15 min. The antimicrobial effect of Umonium^38^ remained for 14 days.

**Supplementary Information:**

The online version contains supplementary material available at 10.1186/s12879-024-09102-9.

## Significance

and impact of the study: The study provides evidence supporting the efficacy of Umonium^38^ as a bactericidal agent against *E. coli* and *P. aeruginosa*, specifying the required concentrations and contact time. Our investigation adds to the existing knowledge by demonstrating the bactericidal activity of Umonium^38^ against *B. pseudomallei* and MRSA. A limitation of the study is that the results presented have been produced under optimal laboratory conditions, and additional studies are required to determine the effectiveness of Umonium^38^ and Virkon^®^ under sub-optimal conditions, including high levels of organic matter or variable pH.

**Supplementary Information:**

The online version contains supplementary material available at 10.1186/s12879-024-09102-9.

## Introduction

Effective disinfection is crucial for infection control as it helps to control potentially hazardous microorganisms, especially in laboratory environments. It is crucial to use validated decontamination protocols to effectively inactivate pathogens as this reduces the likelihood of pathogen exposure, resulting in laboratory-acquired infections and contamination of laboratory and outside environments. The selection of a disinfectant is often based on various factors, including the pathogens to be manipulated, compatibility with laboratory surfaces and efficacy of inactivation [1]. This is particularly important in low-resource settings, where cost, availability, and concentration influence decisions.

Laboratory personnel consistently face significant hazards when exposed to hazardous and potentially lethal pathogens. The routine handling of pathogenic biological agents by laboratory personnel necessitates adherence to stringent safety protocols due to the inherent risk of infection. *P. aeruginosa* is an opportunistic bacterium classified as risk group 2 [2] that frequently causes nosocomial infections, particularly in patients with burn wounds, cystic fibrosis, acute leukaemias, organ transplantation, and intravenous drug addiction [3]. Inadequate infection control protocols can contribute to its persistence. They can survive under various environmental conditions, such as storage tanks, disinfectant solutions, and urinals in hospital environments [4]. According to the Centers for Disease Control and Prevention (CDC), *P. aeruginosa* causes 51,000 healthcare-associated infections in US hospitals annually, with 13% of cases exhibiting multidrug resistance, leading to 440 deaths each year [5]. Similarly, Methicillin-Resistant *Staphylococcus aureus* (MRSA) infection is a significant source of nosocomial and community-associated infections, potentially leading to mortality due to its resistance to conventional beta-lactam antibiotics [6]. The manipulation of *S. aureus* necessitates adherence to Biosafety Level 2 practices and procedures [7]. Specific populations, including athletes, daycare and school children, military personnel residing in barracks, and individuals undergoing inpatient medical care, surgery, or using medical devices, are more susceptible to MRSA infection [8].

*Burkholderia pseudomallei* is a gram-negative bacterium classified as risk group 3 and causes melioidosis infection [9, 10]. Patients infected with this bacterium often experience symptoms that can be easily confused with other diseases, such as tuberculosis [11]. *B. pseudomallei* infection can lead to local infection, bacteremia, pulmonary infection, and disseminated infection, with a mortality rate of approximately 21% [9]. *Escherichia coli* is mostly harmless to humans; however, some strains, such as enterotoxigenic *E. coli* O157:H7 [12, 13], can cause serious illness. These pathogenic strains are known to contaminate food and water sources, leading to symptoms such as diarrhoea and poisoning in people who come into contact with them.

Umonium^38^ has been reported as a broad-spectrum disinfectant for laboratory purposes for a wide range of bacteria, viruses, and fungi. The active ingredient of Umonium^38^ is isopropyl-tridecyl-dimethyl-ammonium, a surfactant that breaks the bonds between water molecules and penetrates deeper into micro-asperities, allowing it to dissolve other molecules [14]. Studies have demonstrated that Umonium^38^ is a highly effective disinfectant against avian influenza virus (AIV) subtype H5N1 and Newcastle disease virus (NDV) [15]. Furthermore, Umonium^38^, when combined with other active compounds, exhibits anti-mycobacterial and antibacterial properties [16]. Umonium^38^ offers several advantages, such as its broad antibacterial properties, relative affordability, and user safety since it contains no carcinogenic or endocrine-disrupting components [14]. It is also compatible with several industrial and equipment surfaces, thanks to its neutral pH, non-flammability, and lack of toxic gas emissions [14].

This study aimed to assess the bactericidal efficacy of various concentrations of Umonium^38^ against four bacterial species: *B. pseudomallei*, *E. coli*, MRSA and *Pseudomonas aeruginosa.* We also examined the bactericidal efficacy of Umonium^38^ and determined its stability over 14 days post-treatment. Furthermore, we aimed to compare the bactericidal efficacy of Umonium^38^ with Virkon^®^, a widely employed and currently available laboratory disinfectant.

## Materials and methods

### Bacterial strains and disinfectants

The bacterial strains used in the study were all clinical isolates either from the American Type Culture Collection (ATCC) or from clinical studies performed in Thailand: *P. aeruginosa* (PA) strain Boston 41,501 (ATCC 27,853), *E. coli* strain Seattle 1946 (ATCC 25,922), MRSA strain S021 (Northeastern Thailand; 2008) and *B. pseudomallei* (BP) strain 1026b [17, 18] (BEI strain NR-9910, Northeastern Thailand; 1993). Two commercial disinfectants, Umonium^38^ (Huckert’s International, Belgium) and Virkon^®^ (Antec International Ltd, United Kingdom), were assessed for effectiveness against these four bacterial strains.

### Bacterial suspension preparation

Bacteria were retrieved from frozen stocks and sub-cultured using selective Ashdown’s agar for *B. pseudomallei* and Columbia agar for *E. coli*, MRSA, and *P. aeruginosa*. The bacteria were incubated at 37 ^o^C for two days before re-subculturing 3–5 colonies on Columbia agar and incubated at 37 ^o^C overnight. A bacterial suspension was formed by emulsifying pure colonies in 20 mL of normal saline solution (NSS). The bacterial turbidity was adjusted to meet the McFarland standard number 7.0, which resulted in an estimated bacterial concentration ranging from 1.0 to 3.0 × 10^9^ CFU/mL (stock concentration), which was used for in vitro testing purposes.

### In vitro bacterial viability testing

**Fig. 1:**
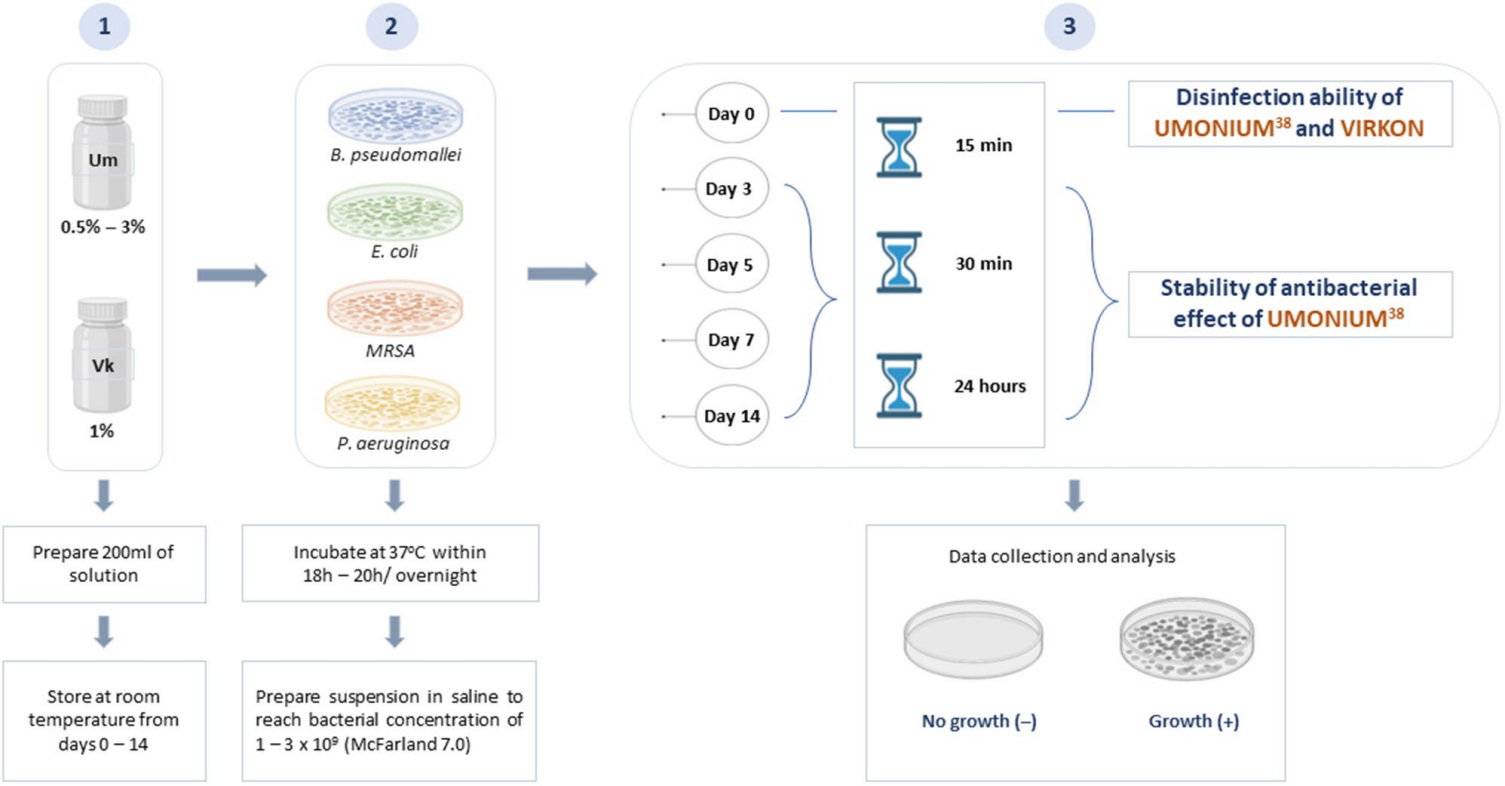
Research objectives and experimental design

Umonium^38^ was diluted in distilled water at concentrations of 0.5%, 1%, 1.5%, 2%, 2.5%, and 3% (v/v) and stored at room temperature (25–35 °C). Concentrations were selected based on manufacturers’ recommendations and practical considerations, including overnight soaking of contaminated materials. A negative control (NC) of 1% (w/v) Virkon^®^ and a positive control (PC) of distilled water were used for each Umonium^38^ concentration (refer to Table 1). To achieve a bacterial concentration in the range of 2–6 × 10^8^ CFU/mL in each tube, 1mL of 1–3 × 10^9^ CFU/mL stock concentration of each isolate was dispensed into tubes containing 4 mL (ratio 1:5) of 0.5–3% concentrations of Umonium^38^ (see Fig. 1). After 15 min, 30 min and 24 h of contact time, 100 µL of each Umonium^38^ concentration, negative control tubes and positive control tubes was removed and spread onto Columbia agar plates, and another 100 µL was added to 3 mL of enrichment tryptone soya broth (TSB). To determine the bacterial viability result on day 0, plates and broth tubes were incubated at 37 °C for 48 h, and the resulting colonies on cultured plates were counted and calculated to CFU/mL. After 48 h of incubation for broth tubes without shaking, use 100 µL to spread onto Columbia agar plate (perform duplicated plates per broth tube). Plates were incubated at 37 °C for 48 h, and viability testing results were read as growth (+) and no growth (-)(see Fig. 1).

**Table 1:** Overview of the disinfectants and the concentration of their active ingredients tested

Stability of Um^38^ testing on	Um concentration	Descriptions	Contact time and viability testing
Day 0, 3, 5, 7 and 14	0.5%	0.5% Um^38^ + bacteria	15, 30 min and 24 h
		0.5% Um^38^ + bacteria	
		0.5% Um^38^ + bacteria	
		1% Virkon + bacteria (NC)	
		DW + bacteria (PC)	
Day 0, 3, 5, 7 and 14	1.0%	1.0% Um^38^ + bacteria	15, 30 min and 24 h
		1.0% Um^38^ + bacteria	
		1.0% Um^38^ + bacteria	
		1% Virkon + bacteria (NC)	
		DW + bacteria (PC)	
Day 0, 3, 5, 7 and 14	1.5%	1.5% Um^38^ + bacteria	15, 30 min and 24 h
		1.5% Um^38^ + bacteria	
		1.5% Um^38^ + bacteria	
		1% Virkon + bacteria (NC)	
		DW + bacteria (PC)	
Day 0, 3, 5, 7 and 14	2.0%	2.0% Um^38^ + bacteria	15, 30 min and 24 h
		2.0% Um^38^ + bacteria	
		2.0% Um^38^ + bacteria	
		1% Virkon + bacteria (NC)	
		DW + bacteria (PC)	
Day 0, 3, 5, 7 and 14	2.5%	2.5% Um^38^ + bacteria	15, 30 min and 24 h
		2.5% Um^38^ + bacteria	
		2.5% Um^38^ + bacteria	
		1% Virkon + bacteria (NC)	
		DW + bacteria (PC)	
Day 0, 3, 5, 7 and 14	3%	3% Um^38^ + bacteria	15, 30 min and 24 h
		3% Um^38^ + bacteria	
		3% Um^38^ + bacteria	
		1% Virkon + bacteria (NC)	
		DW + bacteria (PC)	

*Note* All variations of disinfectant concentration and contact time were tested in triplicate for each bacterium

Um^38^ Umonium^38^

DW Distilled Water

NC Negative Control

PC Positive Control

### Stability of disinfection

To determine the stability of Umonium^38^ over 14 days, the same procedure was followed for preparing the cultures and Umonium^38^ at the six concentrations (i.e., 0.5%, 1%, 1.5%, 2%, 2.5%, and 3%). Viability testing on days 3, 5, 7, and 14 with different contact times of 15 min, 30 min and 24 h was performed on an agar plate and broth as described on day 0. TSB tubes were inoculated in triplicate and incubated at 37 °C without shaking, and culture attempted after 48 h of incubation by collecting 100 µL from TSB tubes to spread onto Columbia agar plates and plates were incubated at 37 °C for 48 h, with growth (+) or no growth (-) recorded for each organism. In addition to the six Umonium^38^ concentrations, a 1% Virkon NC and Distilled water PC were also included. A summary of all the tested disinfectants and concentrations is presented in Table 1.

## Results

### Umonium^38^ and virkon^®^

Umonium^38^ demonstrated potent antimicrobial activity against *P. aeruginosa, E. coli, B. pseudomallei*, and MRSA. At a concentration of 0.5%, Umonium^38^ completely inactivated *P. aeruginosa, E. coli*, and MRSA within 15 min of exposure (Table 2 and Table S1). However, *B. pseudomallei* required 1% Umonium^38^ with contact for 15 min or 0.5% concentration with contact for 24 h to achieve a complete kill (Table 2 and Table S1). While also effective against the four bacterial strains, 1% Virkon^®^ required a contact time of 30 min to achieve comparable outcomes (Table S1).

**Table 2:** Results indicating minimum concentrations of Umonium^38^ and contact times to inactivate *P. aeruginosa*, MRSA, *B. pseudomallei*, and *E. coli* following incubation from day 0–14

Organism	Day 0	Day 3	Day 5	Day 7	Day 14
*B. pseudomallei*	1% (15 min)/0.5% (24 h)	1% (15 min)/0.5% (24 h)	1% (15 min)/0.5% (24 h)	1% (15 min)/0.5% (24 h)	1% (15 min)/0.5% (24 h)
*P. aeruginosa*	0.5% (15 min)	0.5% (15 min)	0.5% (15 min)	0.5% (15 min)	0.5% (15 min)
*E. coli*	0.5% (15 min)	0.5% (15 min)	0.5% (15 min)	0.5% (15 min)	0.5% (15 min)
MRSA	0.5% (15 min)	0.5% (15 min)	0.5% (15 min)	0.5% (15 min)	0.5% (15 min)

### Stability of Umonium^38^ antibacterial efficiency

Umonium^38^ consistently demonstrated its efficacy throughout the experiment by inactivating *P. aeruginosa*, *E. coli*, *B. pseudomallei*, and MRSA to the same extent on day 3 as on day 0. No growth of these bacterial strains was observed on day 5. Although *B. pseudomallei* requires a longer contact time of 30 min to achieve a similar effect, exposure to 0.5% Umonium^38^ for just 15 min resulted in complete inactivation of all four bacterial strains on days 7 and 14, as shown in Table 2 and Table S1. Additionally, no growth of these organisms was observed after 14 days of incubation with 1% Virkon^®^ (Table S1).

## Discussion

This study is the first to determine the most effective Umonium^38^ concentration and duration of contact for inactivation of *P. aeruginosa, E. coli, B. pseudomallei*, and MRSA under optimal conditions. 1% Virkon^®^ was also effective against the four bacterial strains following a 30-minute contact time.

The results presented in this study reflected those of the Umonium^38^ manufacturer (summarised in Table 3) on *P. aeruginosa* and *E. coli* using European Standards EN 1276:2019 and EN 1040:2006 [19]. They reported a contact time of > 10 min, and a 0.5% Umonium^38^ solution resulted in a reduction of over 10^5^ in both bacteria; however, after a 1-minute contact, the reduction reported was < 10^5^ [19]. The report also mentioned that a higher concentration of 2.5% Umonium^38^ required a minimum contact time of only 1 min to achieve the same bactericidal effect as 0.5% Umonium^38^ following 10 min of contact [19]. Similar results were achieved for inactivating *P. aeruginosa* and *E. coli* using 0.5% and 2.5% Umonium^38^ following 15 min of contact following the European Standard EN13697:2001 [16].

**Table 3:** Summary of the manufacturer’s (Huckert’s International, Belgium) antibacterial validation results and the results of studies on *P. aeruginosa* (ATCC 27,853) and *E. coli* (ATCC 25,922). Adapted from [19]. Numbers in bold are the results from this study.

Organism	Concentration (%)	Contact time (min)	Reduction (logs)	Method	Standard
*P. aeruginosa* (ATCC 27,853)	0.5	10	> 5	Dilution/neutralisation	EN 1276
	2.5	1	> 5	Dilution/neutralisation	EN 1276
	0.5	1	< 5	Glass/ PVC	EN 1040
	0.5	10	> 5	Glass/ PVC	EN 1040
	0.5	30	> 5	Glass/ PVC	EN 1040
	2.5	1	> 5	Glass/ PVC	EN 1040
	2.5	10	> 5	Glass/ PVC	EN 1040
	2.5	30	> 5	Glass/ PVC	EN 1040
	**0.5**	**15**	**8**	This study	
*E. coli* (ATCC 25,922)	0.5	10	> 5	Dilution/neutralisation	EN 1276
	2.5	1	> 5	Dilution/neutralisation	EN 1276
	0.5	1	< 5	Glass/ PVC	EN 1040
	0.5	10	> 5	Glass/ PVC	EN 1040
	0.5	30	> 5	Glass/ PVC	EN 1040
	2.5	1	> 5	Glass/ PVC	EN 1040
	2.5	10	> 5	Glass/ PVC	EN 1040
	2.5	30	> 5	Glass/ PVC	EN 1040
	**0.5**	**15**	**8**	This study	

Our results demonstrated that 0.5% Umonium^38^ can effectively inactivate MRSA within 15 min, sustained for 14 days, and underscores its potential for safely and effectively disinfecting equipment surfaces and laboratory environments. MRSA inactivation currently focuses on light utilisation, such as far-UVC LEDs with a wavelength below 240 nm [20, 21] and antimicrobial photodynamic therapy with a porphyrinic formulation [22] for antiseptic purposes on the skin. In addition, the efficacy of octenidine hydrochloride has been assessed for the inactivation of MRSA biofilm formation on medical implants and laboratory equipment within hospital settings [23].

Our study demonstrated that *B. pseudomallei* required 1% Umonium^38^ with contact for 15 min for effective inactivation. Other chemical treatments, heat exposure, autoclaving, and radiation are also effective for *B. pseudomallei* inactivation. Chemical agents, including chlorine dioxide solution [24], pH-adjusted bleach, ethanol solution (70%), quaternary ammonium compounds, and PineSol^®^ [25] have been proven effective. *B. pseudomallei* can be effectively inactivated by heat treatment at 80^o^C for 1 h [26] or 121 ^o^C for 15 min [27]. Exposure to sunlight with wavelengths ranging from 295 to 305 nm can inactivate *B. pseudomallei* concentrations from 10^4^ to 10^6^ CFU/ml in 60 to 180 min [28]. Furthermore, ultraviolet (UV) light with a wavelength of 365 nm, emitting a radiant flux of 90,000 mWs/cm^2^ at a flow rate of 5 L/min, resulted in the inactivation of *B. pseudomallei* 10^6^ CFU/mL [29].

A limitation of the results presented in this study is that it has been performed under optimal conditions, and the results should be interpreted as such. The effectiveness of chemical disinfectants under different background conditions, including pH, and in the presence of significant biological matrices, including culture media or organic contamination, may affect the efficacy of the inactivation of Umonium^38^. Raffo et al. [16] investigated the impact of “clean” and “dirty” conditions on the effectiveness of 0.5% and 2.5% Umonium^38^ for inactivating *P. aeruginosa* and *E. coli*. The “clean” condition involved 0.3 g of bovine serum albumin per liter of water, while the “dirty” condition involved the combination of 3.3 g of bovine serum albumin with 3.0 ml of red blood cells per liter of water. The study found that for both *P. aeruginosa* and *E. coli*. under “clean” conditions, 0.5% Umonium^38^ treatment gave a 4-log_10_ reduction in infectivity after 15 min; however, 60 min was required under “dirty” conditions [16]. Interestingly, 2.5% Umonium^38^ achieved a 4-log_10_ reduction in infectivity of both pathogens after 15-minute exposure for both “clean” and “dirty” conditions [16]. Therefore, additional studies are required to determine the optimal concentrations for the inactivation of *B. pseudomallei* and MRSA under sub-optimal conditions, including organic loads or varied pH.

The results presented here demonstrate the effectiveness of Umonium^38^ and Virkon^®^ for a selected group of bacteria under optimal conditions. When used correctly, Umonium^38^ offers laboratory staff an alternative for effective disinfection and provides an affordable and practical method for routine disinfection. Further work is required to determine the effectiveness of this and other disinfectants under the practical circumstances of everyday use.

### Electronic supplementary material

Below is the link to the electronic supplementary material.

Supplementary Material 1 - Growth of tested pathogens in various concentrations and contact times of Umonium38, 1% Virkon (NC) and distilled water (PC) observed at days 0, 3, 5, 7, and 14.

## Data Availability

All data generated or analysed during this study are included in this published article.
