# Supplementary material for: Effectiveness of Umonium38 against Burkholderia pseudomallei, Escherichia coli, Pseudomonas aeruginosa and Methicillin-Resistant Staphylococcus aureus (MRSA)
Source: BMC Infect Dis. 2024 Feb 16;24:212. doi: 10.1186/s12879-024-09102-9 (PMC10873964; doi:10.1186/s12879-024-09102-9)
Supplement: Supplementary file 1 — Supplementary Material 1 - Growth of tested pathogens in various concentrations and contact times of Umonium38, 1% Virkon (NC) and distilled water (PC) observed at days 0, 3, 5, 7, and 14. [file 12879_2024_9102_MOESM1_ESM.docx]

**S1 Table.**

Growth of tested pathogens in various concentrations and contact times of Umonium^38^, 1% Virkon^®^ (NC) and distilled water (PC) observed at days 0, 3, 5, 7, and 14.

|  | | Bacterial growth over time | | | | | | | | | | | | | | |  |  |
| --- | --- | --- | --- | --- | --- | --- | --- | --- | --- | --- | --- | --- | --- | --- | --- | --- | --- | --- |
|  | | Day 0 | | | Day 3 | | | Day 5 | | | Day 7 | | | Day 14 | | | NC | PC |
| Contact time (mins.) | | 15 | 30 | 24hrs. | 15 | 30 | 24hrs. | 15 | 30 | 24hrs. | 15 | 30 | 24hrs. | 15 | 30 | 24hrs. |  |  |
| Organism | Um^38^ conc. (%) |  |  |  |  |  |  |  |  |  |  |  |  |  |  |  |  |  |
| *B. pseudomallei* | 0.5 | + | + | - | + | + | - | + | + | - | + | + | - | + | + | - | - | + |
|  | 1.0 | - | - | - | - | - | - | - | - | - | - | - | - | - | - | - | - | + |
|  | 1.5 | - | - | - | - | - | - | - | - | - | - | - | - | - | - | - | - | + |
|  | 2.0 | - | - | - | - | - | - | - | - | - | - | - | - | - | - | - | - | + |
|  | 2.5 | - | - | - | - | - | - | - | - | - | - | - | - | - | - | - | - | + |
|  | 3.0 | - | - | - | - | - | - | - | - | - | - | - | - | - | - | - | - | + |
| Starting concentration (CFU/mL) | | 3.10 x 10^8^ | | | 2.36 x 10^8^ | | | 2.90 x 10^8^ | | | 2.81 x 10^8^ | | | 2.83 x 10^8^ | | |  |  |
| *P. aeruginosa* | 0.5 | - | - | - | - | - | - | - | - | - | - | - | - | - | - | - | - | + |
|  | 1.0 | - | - | - | - | - | - | - | - | - | - | - | - | - | - | - | - | + |
|  | 1.5 | - | - | - | - | - | - | - | - | - | - | - | - | - | - | - | - | + |
|  | 2.0 | - | - | - | - | - | - | - | - | - | - | - | - | - | - | - | - | + |
|  | 2.5 | - | - | - | - | - | - | - | - | - | - | - | - | - | - | - | - | + |
|  | 3.0 | - | - | - | - | - | - | - | - | - | - | - | - | - | - | - | - | + |
| Starting concentration (CFU/mL) | | 3.58 x 10^8^ | | | 4.94 x 10^8^ | | | 3.51 x 10^8^ | | | 2.83 x 10^8^ | | | 4.24 x 10^8^ | | |  |  |
| *E. coli* | 0.5 | - | - | - | - | - | - | - | - | - | - | - | - | - | - | - | - | + |
|  | 1.0 | - | - | - | - | - | - | - | - | - | - | - | - | - | - | - | - | + |
|  | 1.5 | - | - | - | - | - | - | - | - | - | - | - | - | - | - | - | - | + |
|  | 2.0 | - | - | - | - | - | - | - | - | - | - | - | - | - | - | - | - | + |
|  | 2.5 | - | - | - | - | - | - | - | - | - | - | - | - | - | - | - | - | + |
|  | 3.0 | - | - | - | - | - | - | - | - | - | - | - | - | - | - | - | - | + |
| Starting concentration (CFU/mL) | | 1.88 x 10^8^ | | | 1.94 x 10^8^ | | | 2.10 x 10^8^ | | | 2.53 x 10^8^ | | | 2.34 x 10^8^ | | |  |  |
| *S. aureus,* MRSA | 0.5 | - | - | - | - | - | - | - | - | - | - | - | - | - | - | - | - | + |
|  | 1.0 | - | - | - | - | - | - | - | - | - | - | - | - | - | - | - | - | + |
|  | 1.5 | - | - | - | - | - | - | - | - | - | - | - | - | - | - | - | - | + |
|  | 2.0 | - | - | - | - | - | - | - | - | - | - | - | - | - | - | - | - | + |
|  | 2.5 | - | - | - | - | - | - | - | - | - | - | - | - | - | - | - | - | + |
|  | 3.0 | - | - | - | - | - | - | - | - | - | - | - | - | - | - | - | - | + |
| Starting concentration (CFU/mL) | | 1.17 x 10^8^ | | | 2.21 x 10^8^ | | | 2.10 x 10^8^ | | | 2.34 x 10^8^ | | | 2.47 x 10^8^ | | |  |  |

+ Growth Um^38^ Umonium^38^

- No growth CFU/mL Colony Forming Units/mL

NC Negative Control

PC Positive Control
